# Effects of Electrospinning Parameter Adjustment on the Mechanical Behavior of Poly-ε-caprolactone Vascular Scaffolds

**DOI:** 10.3390/polym14020349

**Published:** 2022-01-17

**Authors:** Anna A. Dokuchaeva, Tatyana P. Timchenko, Elena V. Karpova, Sergei V. Vladimirov, Ilya A. Soynov, Irina Y. Zhuravleva

**Affiliations:** 1 Institute of Experimental Biology and Medicine, E. Meshalkin National Medical Research Center of the RF Ministry of Health, 15 Rechkunovskaya St., Novosibirsk 630055, Russia; t_timchenko@meshalkin.ru (T.P.T.); vladimirov_s@meshalkin.ru (S.V.V.); i_soynov@mail.ru (I.A.S.); zhuravleva_i@meshalkin.ru (I.Y.Z.); 2 Center of Spectral Investigations, Group of Optical Spectrometry, N.N. Vorozhtsov Novosibirsk Institute of Organic Chemistry SB RAS, 9 Lavrentiev Avenue, Novosibirsk 630090, Russia; karpovae@nioch.nsc.ru

**Keywords:** electrospinning, mechanical testing, polycaprolactone, tissue engineering, vascular scaffolds

## Abstract

Electrospinning is a perspective method widely suggested for use in bioengineering applications, but the variability in currently available data and equipment necessitates additional research to ascertain the desirable methodology. In this study, we aimed to describe the effects of electrospinning technique alterations on the structural and mechanical properties of (1,7)-polyoxepan-2-one (poly-ε-caprolactone, PCL) scaffolds, such as circumferential and longitudinal stress/strain curves, in comparison with corresponding properties of fresh rat aorta samples. Scaffolds manufactured under different electrospinning modes were analyzed and evaluated using scanning electronic microscopy as well as uniaxial longitudinal and circumferential tensile tests. Fiber diameter was shown to be the most crucial characteristic of the scaffold, correlating with its mechanical properties.

## 1. Introduction

Currently, electrospinning is one of the most popular methods in constructing tissue-engineered vessels, and over nine thousand articles have been dedicated to this sphere of graft production. This method is highly applicable to tissue construction because of its variability: the availability of a large number of polymers and techniques to choose from allows researchers to obtain the desirable composition, size, and architecture of the resultant fibers by regulating specific parameters. These qualities characterize the durability, permeability, cytocompatibility, and biodegradability of the scaffolds in vitro and in vivo [1]. One of the most significant advantages of electrospun scaffolds is the resemblance between the natural extracellular matrix (ECM) and the nanofibers [2], which are much smaller in diameter than the adhering cells, implying the formation of a suitable microenvironment for cell repopulation [3,4,5]. All of these features result in an immunologically neutral temporary implant that functions as a host site for autologous cells and a growth factor-containing system, which will be replaced, in advance, by functionally sustainable autologous tissues.

The modification of the process or material parameters is the standard approach for the adaptation of fibers and porosity to a specific purpose.

Polymer concentration has a direct impact on solvent evaporation and solution viscosity. If the polymer concentration is low, electrospraying is likely to be performed instead of electrospinning [6]. Additionally, under the same conditions, a solution with high polymer concentration will provide fibers of a larger diameter than a more diluted solution [7].

The molecular weight of the polymer characterizes its chain length, which determines the number of polymer chain entanglements within the solution. The number of chain entanglements affects the solution viscosity and surface tension. As a result, polymers with higher molecular weights produce more homogenous and continuous fibers [8]. The elongation of the polymer chains also prevents the breaking of the jet when it is pulled towards the collector [9].

Solution conductivity affects the tensile force applied to the jet, which is placed in the electric field and is determined by the choice of solution composition. Substrate conductivity can be adjusted by including additional salt or ionic components in the solution, which will decrease the diameter of the emitted fiber [10,11,12].

One of the key points in the electrospinning technique is the applied voltage; however, this is also one of the most debated features, as research groups have reported varying findings on its impact on fiber diameter [12,13,14]. Some researchers [15] have suggested that the significance of the applied voltage depends on the composition of the working solution and setup mode, which is a reasonable statement because polymers have diverse molecular masses, conductivities, and chain lengths.

The feed rate characterizes the speed and force with which the jet of the polymer solution is ejected from the nozzle, and it affects the obtained fiber size and shape. A high feed rate results in fibers of larger diameter and increases the risk of defects such as ribbon-like structures and spherical drops, while a low feed rate exerts the opposite effect up to jet interruption. For this parameter, the ratio of the feed rate to the applied voltage should be taken into account, as it defines the amount of polymer supplied to the jet that is acceptable for the flow rate provided by the electric field [16,17]. 

Because there are numerous types of electrospinning setups, collector and spinneret type and speed may or may not be adjustable in specific cases; regardless, both characteristics are important for fiber alignment and form as well as architecture. The pore size, fiber layout, and overall scaffold pattern can be tailored by changing the collector speed, shape, and texture [18,19].

The nozzle tip to collector distance influences the morphology and size of the fiber. An increase in this distance results in thinner filaments, when the opposite may result in nanobead coating owing to incomplete solvent evaporation [20].

To date, one of the reported obstacles in the construction of electrospun vascular scaffolds is the lack of materials with appropriate mechanical properties. Some authors have indicated that the most resorbable polymers have a high Young’s modulus and low failure stress, which also means that they are excessively stiff and inelastic [21].

Poly-ε-caprolactone ((1,7)-polyoxepan-2-one, PCL) is a widely known aliphatic biodegradable polyester that is synthesized by the ring-opening polymerization of ε-caprolactone [22]. PCL is a biocompatible, accessible, durable polymer with a slow degradation rate; it is more compliant to native vessels than Dacron and PTFE and is used mainly for large-diameter grafts [23,24,25,26]. PCL scaffolds demonstrate good cell adhesion; furthermore, the chemical structure of the polymer determines its degradation into caproic acid, which can be metabolized by the recipient’s body, consequently increasing the chances of effective cell repopulation. Scientists have noted strength among its advantages and stiffness among its flaws. Several attempts have been made to improve the physical characteristics of PCL by chain elongation, coating, copolymerization, and molecular mass alteration [21,27,28,29].

Although electrospinning techniques and PCL properties have been well studied, there is variation in available data among research groups because of the diverse choices in polymer molecular mass, solution composition, and setup construction. The biomechanics of scaffolds are usually investigated in uniaxial tensile tests in one direction relative to the axis of the obtained scaffolds [30,31,32]. In this study, we aimed to describe the characteristics of different PCL grafts, manufactured under otherwise identical conditions, including the electrospinning setup (NANON 01-B) and technique, source material, solvent choice, polymer chain length, and ambient factors and evaluated the effect of particular parameters on fiber and matrix structure and on integral mechanical properties of the produced scaffolds. 

## 2. Materials and Methods

### 2.1. Rat Aorta Collection

All experimental procedures were carried out in accordance with EU Directive 2010/63/EU for animal experiments and were approved by the Ethics Committee of E. Meshalkin National Medical Research Center. Five 12-month-old male Wistar rats (700 g, *n* = 5) were euthanized with an overdose of sevoflurane. Abdominal aorta fragments of 25-mm length were excised via abdominal access and rinsed in 0.9% NaCl, and excessive moisture was removed with gauze.

### 2.2. Polymer Composition

Pellets (3-mm diameter) of (1,7)-polyoxepan-2-one (ε-polycaprolactone) with a molecular mass (Mn) of 80 kDa (cat. № 440744) were obtained from Sigma-Aldrich (Sigma-Aldrich Co., St. Louis, MO, USA) and dissolved in pure chloroform (Vekton, Saint Petersburg, Russia) under constant shaking at 37 °C. Each solution was prepared separately on the date of the experiment. In this study, we used PCL solutions of 5%, 8%, 10%, 12%, 14%, and 16% by mass (Table 1).

### 2.3. Vascular Scaffold Fabrication

Tubular scaffolds were manufactured using NANON 01-B electrospinning setup (MECC Inc., Fukudo Ogori-shi, Japan) on an 8-mm rod rotary collector under set up modes, as shown in Table 1.

### 2.4. Scanning Electronic Microscopy (SEM)

SEM imaging of tissue specimens was performed using a SU1000 FlexSEM II scanning electron microscope (Hitachi, Tokyo, Japan). The specimens were fixed appropriately on a specimen stub using conductive tape. Sample observation was performed using a backscattered electron detector at an electron beam energy of 15 keV and a pressure of 30 Pa. Five observation fields were selected for every specimen and were examined at 100×, 250×, 450×, 700×, 800×, and 1000× magnification. The fiber and pore sizes were measured using a FlexSEM1000 operating program.

### 2.5. Mechanical Properties Evaluation

For mechanical tests, 25 mm (length) × 10 mm (width) rectangular fragments were cut out of obtained scaffolds (*n* = 27), placed in the grips of the device, and stretched longitudinally and circumferentially until failure at an extension rate of 10 mm/min using an ESM 303 L tester (Mark-10 Corporation, Copiague, NY, USA) and a computer-linked force gauge (0–100 N). Sample thickness (mm) was measured using a digital thickness gauge (Mitutoyo, Kawasaki, Japan) at three points of each fragment. All samples were tested dry, without any preconditioning.

Strength was evaluated as failure stress (*σ*, MPa) (Equation (1)):

(1) $$\sigma =\frac{Fmax}{S}$$

where *σ* is the failure stress, *Fmax* is the peak load, and *S* is the cross-sectional area of the sample.

Failure strain (Δ*Ε*, %) was calculated using (Equation (2)):

(2) $$\Delta E=\frac{Lmax-L0}{L0}\times 100$$

where Δ*Ε* is the failure strain, *L*0 is the initial sample length (mm) equal to the distance between the grips, and *Lmax* is the maximal deformation (mm). 

Stiffness was evaluated using Young’s modulus (ε, MPa) (Equation (3)):

(3) $$\varepsilon =\frac{\sigma }{\Delta E}$$

where *σ* is the failure stress and Δ*Ε* is the failure strain Δ*Ε* = (*Lmax* − *L*0)/*L*0.

### 2.6. Statistical Analysis

Statistical analyses were performed using software Stata 14 (StataCorp LP, College Station, TX, USA). Categorical variables are presented as frequencies and percentages, and continuous variables are presented as mean ± standard deviation. Univariate and multivariate linear regression analyses were used to identify predictor variables. Statistical significance was set at *p* < 0.05.

## 3. Results

Numerical data of the described experiments are presented in Table 2.

**Figure 1:**
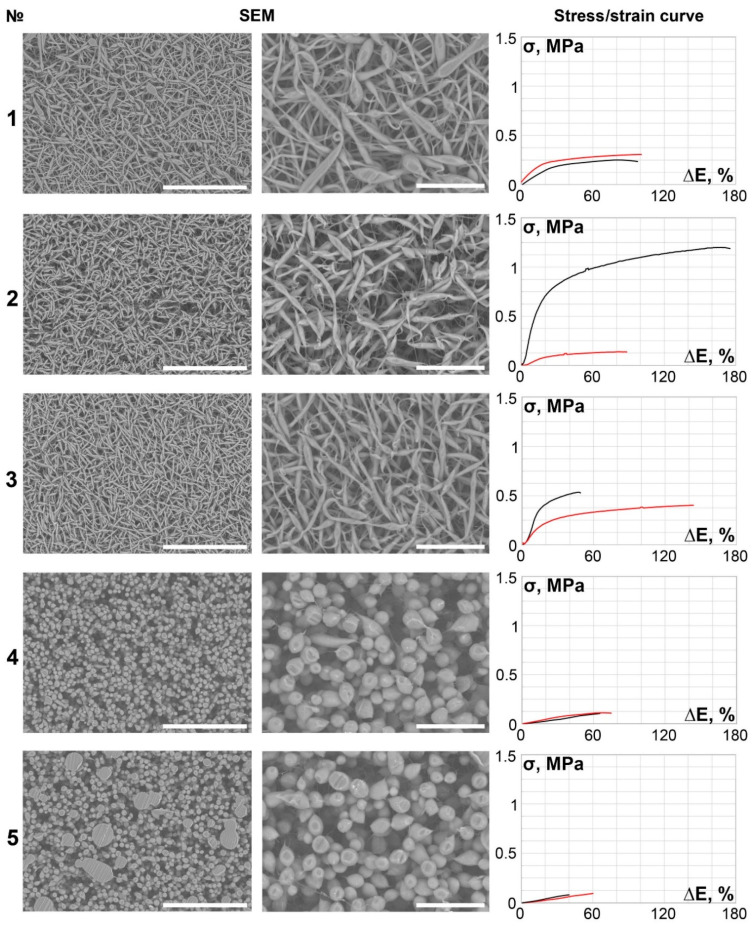
SEM images (×250 magnification, scale bar 200 µm and ×700 magnification, scale bar 50 µm) and stress/strain curves of tested 5% PCL samples. Red curves reflect stress/strain plotting in the axial direction; black curves correspond to circumferential stretching.

Scaffolds obtained from the 5% PCL solution differed significantly from the other samples in terms of microstructure. The scaffolds comprised a mixture of short, disorganized spindle-shaped structures and fibers when using a larger diameter nozzle (Figure 1, № 1–3).

However, when using a smaller diameter nozzle (27 G), the structures become 9.42–16.7-µm spherical droplets, often called “nanobeads” or “microbeads” in literature [33,34] (Figure 1, № 4, 5).

This effect may be explained by the low solvent concentration and small needle-tip section area, which causes electrospraying instead of electrospinning because the solution has low viscosity and drops easily detach from the nozzle tip. Pore size varies from an average of 11.34 to 16.9 µm, defining the scaffolds as moderately porous overall. 

We encountered certain difficulties in describing the pore size in samples made with solutions of low polymer concentration. Because of the particle shape (nanobeads and short oblong fragments) and scattered layout, it was difficult to determine the pore margins.

Scaffolds No. 4 and 5 of 5% PCL group appeared to be the least durable both circumferentially and longitudinally, but the low Young’s modulus and the flatter beginning of the stress/strain curve indicated that these scaffolds were less stiff.

**Figure 2:**
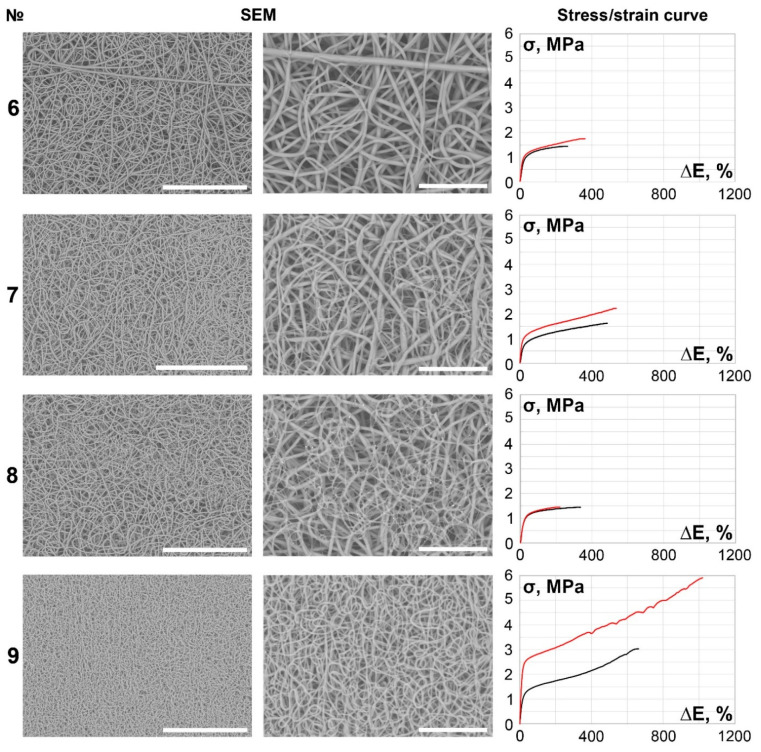
SEM images (×250 magnification, scale bar 200 µm and ×700 magnification, scale bar 50 µm) and stress/strain curves of tested 8% PCL samples. Red curves reflect stress/strain plotting in the axial direction; black curves correspond to circumferential stretching.

**Figure 3:**
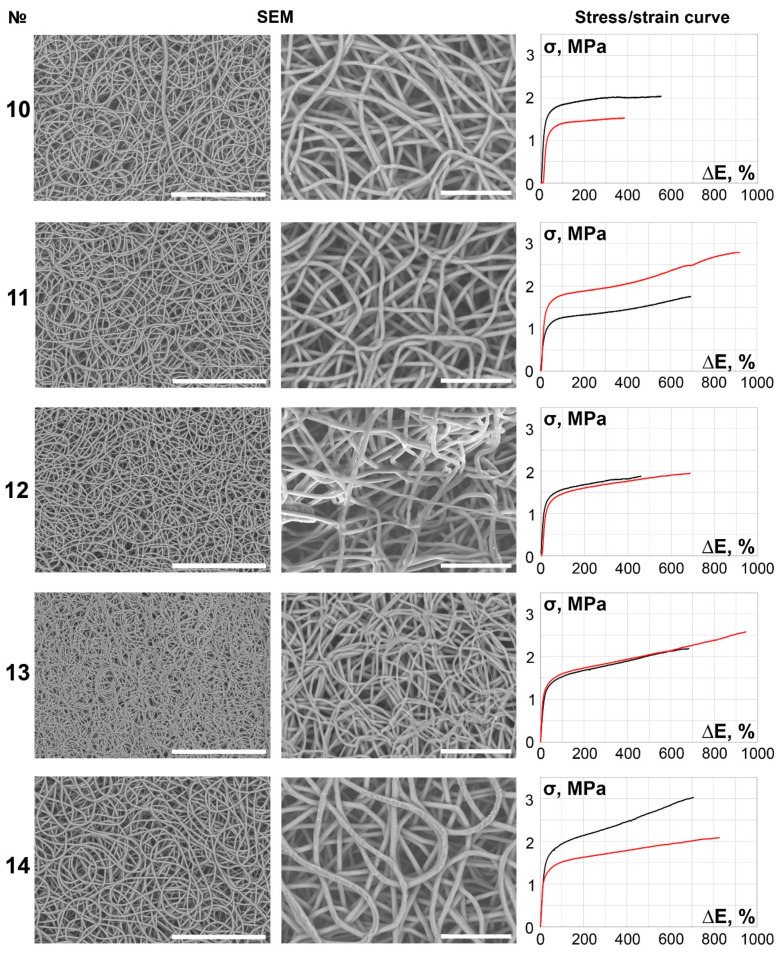
SEM images (×250 magnification, scale bar 200 µm and ×700 magnification, scale bar 50 µm) and stress/strain curves of tested 10% PCL samples. Red curves reflect stress/strain plotting in the axial direction; black curves correspond to circumferential stretching.

**Figure 4:**
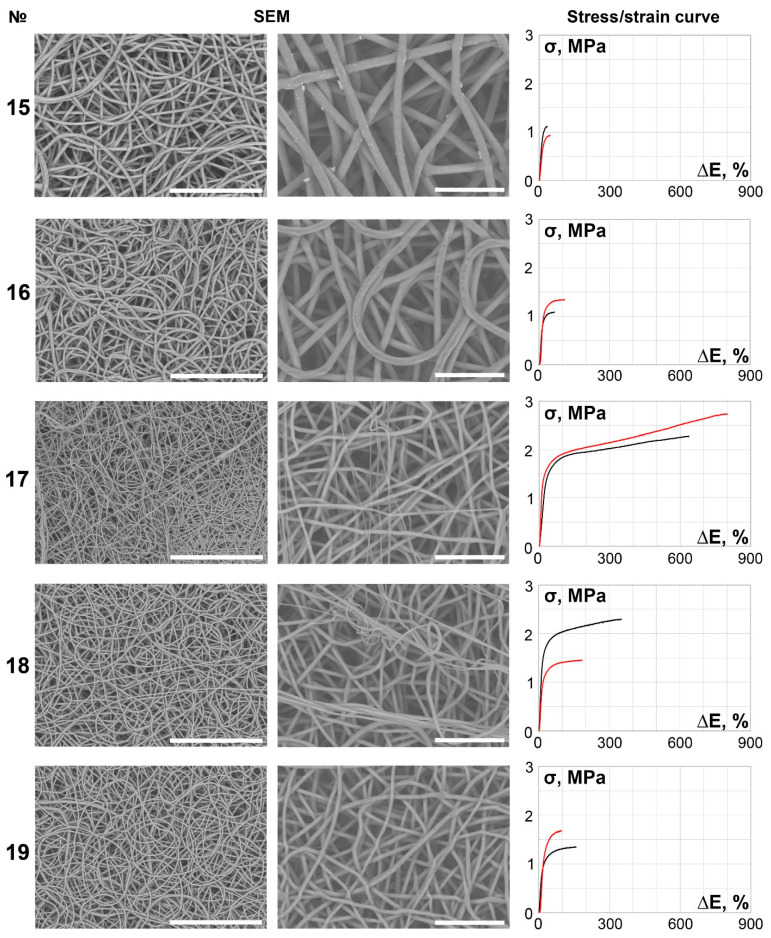
SEM images (×250 magnification, scale bar 200 µm and ×700 magnification, scale bar 50 µm) and stress/strain curves of tested 12% PCL samples. Red curves reflect stress/strain plotting in the axial direction; black curves correspond to circumferential stretching.

**Figure 5:**
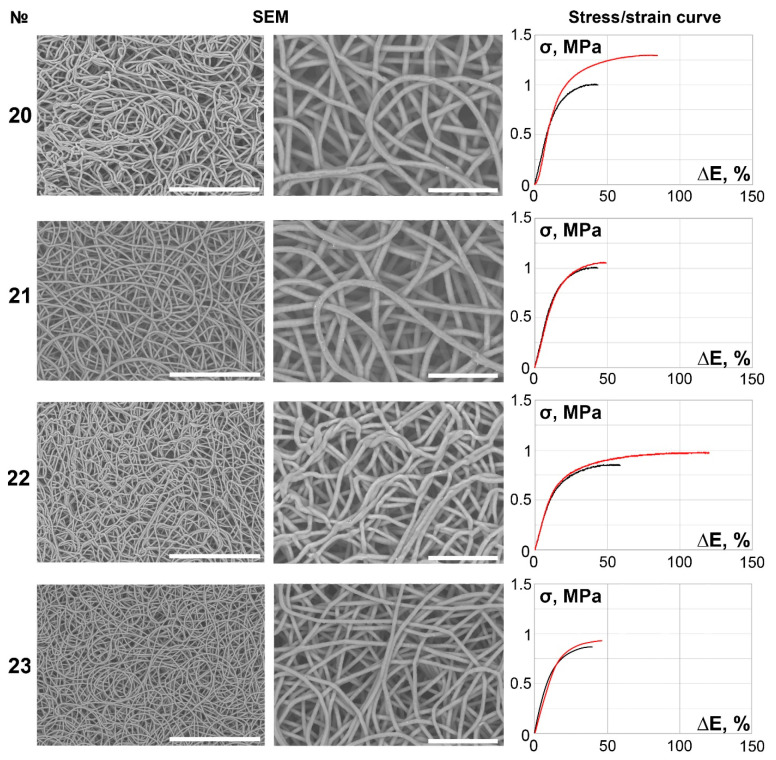
SEM images (×250 magnification, scale bar 200 µm and ×700 magnification, scale bar 50 µm) and stress/strain curves of tested 14% PCL samples. Red curves reflect stress/strain plotting in the axial direction; black curves correspond to circumferential stretching.

**Figure 6:**
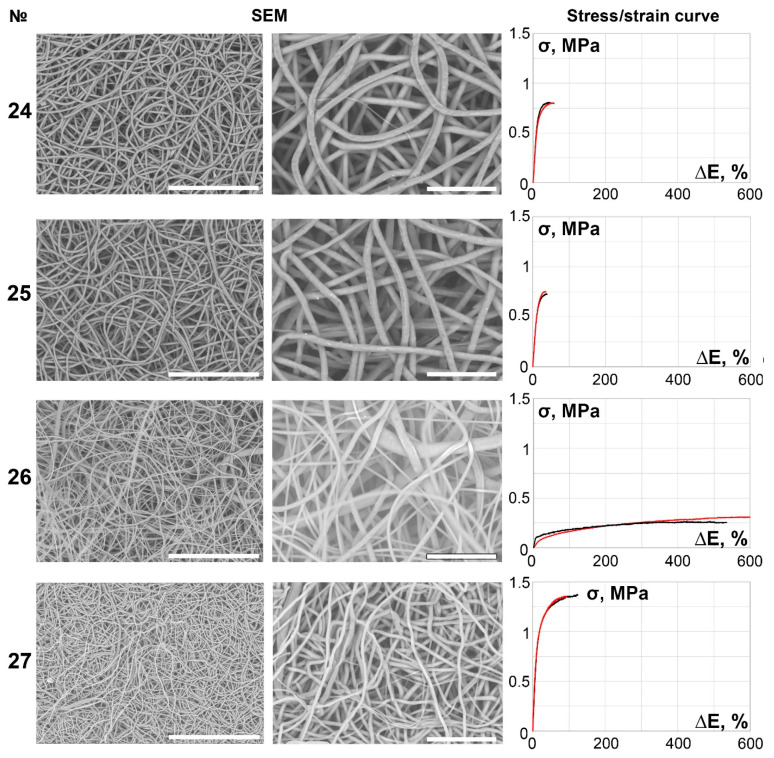
SEM images (×250 magnification, scale bar 200 µm and ×700 magnification, scale bar 50 µm) and stress/strain curves of tested 16% PCL samples. Red curves reflect stress/strain plotting in the axial direction; black curves correspond to circumferential stretching.

The main part of the scaffolds (samples 6–27) demonstrated continuous, smooth, primarily isotropic fibers assembled in a dense looped pattern (Figure 2, Figure 3, Figure 4, Figure 5 and Figure 6).

It is notable that specimens produced under lower spinneret speed modes tended to be more anisotropic (Table 2, Figure 1, Figure 2, Figure 3, Figure 4, Figure 5 and Figure 6), regardless of the polymer solution concentration, as seen in samples 1, 5, 9, and 12. However, an increase in the spinneret speed results in an increase in the pore size and a decrease in the fiber diameter, consequently increasing the circumferential stiffness. The use of a smaller nozzle decreased the fiber diameter and pore size. Higher applied voltages and lower polymer concentrations increased the number of fiber-to-fiber connections and decreased the stiffness of the scaffold.

We noted that solutions with higher polymer concentrations require lower feed rates, especially when a smaller nozzle is used. If the feed rate is too high and the solution is ejected into the electrostatic field with excessive force, it results in an additional solution volume at the forming Taylor cone, leading to drop collapse and artifacts, similar to those obtained in the case of insufficient solution viscosity. An increase in the collector rotation speed increased the fiber size and decreased the pore diameter.

Among 8% PCL samples, vessel № 9, which demonstrates the maximum value of failure strain axially and second highest value longitudinally, differed from others, as it also had the finest fibers and one of the smallest average pore sizes. This scaffold was manufactured with the lowest polymer concentration solution that provided continuous fibers using a smaller nozzle. This supports our observation that, regardless of the fiber size, scaffolds with larger pores appeared to demonstrate higher rigidity in accordance with the strain/stress curves.

Ambient parameters play a significant role in electrospinning. An increase in the indoor room temperature influences the fluidity of the solution, which results in stock dripping, jet interruption, fiber anisotropy, and droplet artifacts on the collector. The same effect is caused by an increase in air humidity, which can be explained by the slower solvent evaporation. We detected small droplet artifacts and jet interruption during electrospinning process at humidity level of 25%. An increase in air humidity relatively increased the number and size of such artifacts. At the mark of 27% the electrospinning process could not be performed, as jet failed to form and large drops of working solution were found on the collector. As the environmental parameters change, the setup parameters, including applied voltage and feed rate, should be modified correspondingly.

According to multivariable linear regression analysis, an increase in fiber diameter caused a decrease in longitudinal (*p* = 0.018) and circumferential (*p* = 0.049) strength. The main tendency showed that thinner fibers also resulted in the construction of scaffolds with higher failure strain, but no statistically significant data were obtained. The pore diameter correlated with the polymer solution concentration (*p* = 0.038), and an increase in PCL concentration by mass increased pore size.

**Figure 7:**
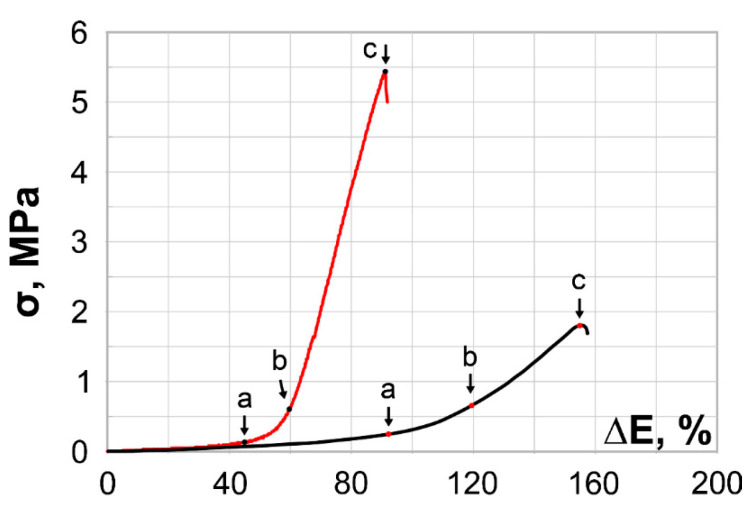
Stress/strain curves of tested rat aorta samples. Elastic limit (a), yield point (b) and ultimate stress point (c). Red curves reflect stress/strain plotting in the axial direction; black curves correspond to circumferential stretching.

The presented stress/strain plots demonstrate the mechanical behavior of the rat abdominal aorta (Figure 7) in comparison with the obtained PCL scaffolds.

## 4. Discussion

Scaffold stress/strain features in the circumferential and longitudinal axes were evaluated on the basis of the analysis of full strain/stress curves, in comparison with fresh rat aorta samples. Our choice of species was based on the consideration that rats are most likely to be used as model animals in further in vivo experiments.

Pore size in combination with fiber thickness characterizes the proportion between the main mass and the polymer surface, which is important for cell migration within the implanted prosthesis and therefore its degradation rate and the quality of cell repopulation. Larger pores result in more types of cells accessing the polymer scaffold as well as faster cell migration and biodegradation. In our case, the preferable pore size was 20–50 µm, according to the cell pool to which the graft will be administered. 

Some authors avoid reporting specific data while describing the electrospinning process, presenting ranges of values or partial information [31,32,35,36] for significant parameters such as polymer concentration, nozzle diameter, collector motion speed, and mixed solvent proportion. According to our findings, these characteristics are crucial for fiber formation. As shown above, highly viscous solutions require a higher applied voltage, lower feed rate, or a wider nozzle opening to produce sustainable fibers.

Eichorn and Sampson reported that thinner fibers seem to have more fiber-to-fiber contacts per unit length [37], but we noticed the same tendency in specimens collected under higher applied voltages (samples 7, 12, and 22). Suwantong mentioned that the appearance of an interconnected network of fibers also depends on solvent volatility and solvent evaporation [38], which was confirmed by our results, as fibers produced from thicker polymer solutions are more likely to interlace than merge. Samples with a larger number of fiber-to-fiber connections were more stretchable and durable than other specimens within their group.

All the electrospinning process characteristics are interrelated and function as a complete system; thus, some of the processing modes cannot be reproduced exactly for all PCL concentrations. In particular, an applied voltage of 16 kV was not efficient for solutions containing 14% and 16% PCL in pure chloroform. As the jet could not form under insufficient tension, the drop of the working solution dried at the nozzle tip without formation of the Taylor cone. In addition, the 5% PCL solution when exposed to a stronger electrostatic field did not form a stable jet either, resulting in large flat artifacts on the collector.

The less stiff and most durable samples were collected from 8% and 10% PCL solutions under modes with low collector and spinneret speeds, but no direct correlation with their average pore size was found. The elongation and strength characteristics of these samples may depend on the number of fiber-to-fiber interconnections and adhesion. 

Yördem et al. [15] reported that with the use of high concentration solutions, the applied voltage has less impact on fiber formation as long as it is sufficient for jet formation. We found that at a polymer concentration of 16%, the fiber thickness depended less on the applied voltage or feed rate and more on the nozzle size, spinneret speed, and collector rotation rate. Owing to the construction limitation of our setup, we had reduced opportunity to adjust the feed rate in high-concentration solutions, as tube-to-nozzle connections were disconnected from the gained pressure. Application of a higher voltage caused the jet to adhere to the side of the collector.

The nozzle cleaning rate is a parameter rarely mentioned in the literature, possibly owing to various constructions of electrospinning setups or the lack of such an option. While using a vertical-type setup, we noted that occasional wiping of the needle tip prevented artifact formation and jet splitting and removed dried pieces of high-concentration solutions from the tip. It also allowed us to obtain several scaffolds from low-concentration solutions (5% and 8%) because of the high fluidity of these solutions caused additional volume of stock to leak down the needle, enlarging the Taylor cone until it became large enough to form a drop and collapsed on the collector. By cleaning the needle tip every 30 s, we collected the excessive solution before it reached the necessary mass and volume to form a drop and collapse. 

O’Connor et al. [39] confirm that electrospinning process requires complex approach and indicate a similar set of significant parameters, including flow rate, applied voltage, collector distance and collection speed. They also suggest that collector rotation speed has more impact on fiber morphology and overall scaffold structure. Our scaffolds were obtained under lower collector rotation speed, and within our range of samples, no correlation between collector rotation speed and fiber alignment was found. It is also notable that setup construction described in that study includes a stationary injection unit and a rotating and traversing mandrel collector. In this case the polymer jet is ejected horizontally, and excessive volume of stock is likely to drop aside of the collector. Unlike, NANON-B has a traversing spinneret and a rotating collector under the needle tip, so the jet is ejected downwards, which may result in changes in overall surface pattern, fiber anisotropy and artifacts if excessive stock volume travels along the forming jet or drips down on the collector surface. We suggest that significance of certain parameters, such as flow rate and spinneret/collector speed may vary depending on the setup construction.

We should note the difference in behavior between PCL scaffolds and natural tissues. During tensile tests, native vessels undergo several phases, as shown in Figure 7: elastic (0–a), transitional (a–b), and collagen (b–c). This behavior is explained by the complex nature of a vessel, where fibers with different biomechanical properties are present. Collagen and elastin fibers vary in morphological organization, strength, and mechanical resilience and reach the point of irreversible or plastic deformation at different times during a common tensile test [40,41]. Therefore, the period between the yield stress point to ultimate stress point on the diagram can be determined as the period during which irreversible changes in tissue begin. Thus, the concepts of elastic, transitional, and collagen phases are not quite transferable to homogenous polymeric artificial scaffolds.

According to the stress/strain curves, all obtained PCL samples were characterized by a lack of an elastic phase and rapid stress gain from the beginning of the test, unlike rat aorta fragments. This is the main disadvantage of Dacron and PTFE, as they are considered unsuitable for manufacturing small vascular prostheses: the excessive stiffness of the prosthesis leads to poor compliance between the original and artificial vessels and may result in intimal hyperplasia and thrombosis. In the case of small-diameter vessels, such side effects cause occlusion and consequent total dysfunction [42].

None of the obtained samples resembled the native rat aorta in terms of mechanical properties. Although the Young’s modulus of several electrospun vessels appeared to be lower than that of native tissues, mechanical behavior characterizes these electrospun vessels as stiff and sub-optimal for grafting for the intended purposes. The scaffolds were made with 8% and 10% PCL solutions were most applicable for in vivo implantation according to their pore size, fiber diameter, Young’s modulus, and biomechanical behavior. The strength properties of specimen No. 9 had relatively sufficient resemblance to those of the native rat aorta samples, compared to other scaffold specimens, but it also has thin fibers, implying that it has fast biodegradation properties and small pores, which decrease its permeability for desirable cell types.

## 5. Conclusions

In this study, we confirmed the parameters that are crucial for the electrospinning process and described their significance. The samples that meet graft requirements for pore size demonstrate non-conforming biomechanical behavior. The pore size can be increased by increasing the polymer solution concentration, increasing the spinneret motion, and decreasing the collector rotation speed. The strength of the scaffold increased with decreasing pore size and an increase in the number of fiber-to-fiber connections. The fiber thickness can be increased by using a larger nozzle diameter, slower spinneret speed, and rapid collector rotation rate. Fiber anisotropy can be achieved by decelerating the spinneret or collector. Additionally, ambient parameters have considerable impact and should be taken into account while adjusting the electrospinning process.

## Figures and Tables

**Table 1:** Electrospinning setup parameters, by polymer solution concentration.

%	№	Needle	Applied Voltage, kV	Feed Rate, mL/h	Collector Speed, rpm	Spinneret Speed, rpm	Tip to Collector Distance, cm	Cleaning Interval, s	Solution Volume, mL
5%	1	22G	16	0.5	300	100	15	30	0.5
	2		16	0.5	300	150	15	30	1
	3		16	0.5	250	150	15	30	1
	4	27G	16	0.5	300	150	15	20	1
	5		16	0.5	250	100	15	20	1
8%	6	22G	16	0.4	300	150	15	30	1
	7		23	0.3	150	100	15	59	0.5
	8	27G	16	0.5	300	150	15	59	1
	9		16	0.5	250	100	15	59	1
10%	10	22G	16	0.5	300	100	15	59	0.5
	11		16	0.5	300	100	14	59	0.5
	12		23	0.3	150	100	15	30	0.5
	13	27G	16	0.5	300	100	15	59	1
	14		16	0.5	300	150	15	59	1
12%	15	22G	17	0.4	300	150	15	30	1
	16		20	0.6	250	200	15	30	0.6
	17	27G	17	0.4	300	150	15	30	0.7
	18		16	0.4	250	150	15	30	0.6
	19		15	0.4	250	100	15	30	0.5
14%	20	22G	20	0.6	250	200	15	30	1
	21		18	0.4	300	150	15	30	0.5
	22		23	0.3	150	100	15	30	0.3
	23	27G	20	0.3	300	150	15	30	0.4
16%	24	22G	23	0.3	150	100	15	30	0.3
	25		20	0.3	300	150	15	30	0.4
	26	27G	20	0.3	300	150	15	30	0.4
	27		20	0.3	250	100	15	20	0.5

**Table 2:** Structural and biomechanical characteristics of obtained PCL samples.

Concentration by Mass, %	№	Fiber Diameter, µm	Pore Diameter, µm	Failure Strain, %		Failure Stress, MPa		E Mod, MPa	
				Axial	Circumferential	Axial	Circumferential	Axial	Circumferential
5	1	4.182 ± 0.72	11.34 ± 0.8	98.43 ± 11.03	95.48 ± 8.82	0.31 ± 0.03	0.31 ± 0.03	0.32 ± 0.02	0.32 ± 0.01
	2	4.688 ± 0.25	14.85 ± 1.3	87.13 ± 9.12	174.26 ± 16.69	0.17 ± 0.02	1.21 ± 0.09	0.19 ± 0.01	0.69 ± 0.03
	3	4.4 ± 0.46	13.32 ± 1.15	47.65 ± 3.87	142.61 ± 12.07	0.54 ± 0.04	0.41 ± 0.03	1.13 ± 0.09	0.29 ± 0.01
	4	12.21 ± 0.46	16.9 ± 0.93	74.02 ± 5.43	94.40 ± 6.53	0.12 ± 0.01	0.19 ± 0.01	0.16 ± 0.01	0.30 ± 0.02
	5	12.46 ± 0.38	15.91 ± 1.37	59.45 ± 2.61	39.38 ± 1.38	0.17 ± 0.02	0.09 ± 0.01	0.28 ± 0.01	0.22 ± 0.01
8	6	2.46 ± 0.24	14.48 ± 0.79	362.20 ± 31.25	264.96 ± 19.62	1.75 ± 0.09	1.44 ± 0.07	0.48 ± 0.03	0.54 ± 0.05
	7	3.02 ± 0.51	10.08 ± 0.56	536.42 ± 35.93	486.60 ± 27.95	2.22 ± 0.17	1.62 ± 0.11	0.41 ± 0.04	0.33 ± 0.01
	8	2.15 ± 0.14	8.49 ± 0.48	219.45 ± 20.86	334.91 ± 22.7	1.46 ± 0.11	1.45 ± 0.08	0.66 ± 0.03	0.43 ± 0.04
	9	1.4 ± 0.03	6.99 ± 0.31	1019.15 ± 79.01	661.82 ± 53.98	5.46 ± 0.38	3.03 ± 0.23	0.54 ± 0.05	0.46 ± 0.05
10	10	3.32 ± 0.27	7.78 ± 0.93	378.75 ± 17.18	553.01 ± 37.34	1.53 ± 0.16	2.05 ± 0.18	0.40 ± 0.02	0.37 ± 0.02
	11	3.34 ± 0.18	5.87 ± 0.75	917.42 ± 51.95	692.0 ± 64.82	2.79 ± 0.21	1.76 ± 0.12	0.30 ± 0.01	0.25 ± 0.01
	12	2.74 ± 0.13	11.26 ± 0.8	668.14 ± 22.78	459.62 ± 39.76	1.95 ± 0.2	1.88 ± 0.1	0.28 ± 0.01	0.41 ± 0.03
	13	2.15 ± 0.13	14.75 ± 1.01	943.76 ± 66.23	681.75 ± 59.21	2.58 ± 0.16	2.19 ± 0.16	0.27 ± 0.01	0.32 ± 0.03
	14	4.75 ± 0.15	36.5 ± 1.85	825.93 ± 72.97	705.90 ± 62. 19	2.09 ± 0.13	3.03 ± 0.25	0.25 ± 0.01	0.43 ± 0.03
12	15	7.8 ± 1.07	42.29 ± 2.31	45.38 ± 3.33	32.93 ± 1.12	0.93 ± 0.01	1.12 ± 0.09	2.06 ± 0.16	3.41 ± 0.27
	16	5.4 ± 0.17	39.42 ± 1.6	106.85 ± 9.32	64.04 ± 4.09	1.35 ± 0.14	1.09 ± 0.09	1.26 ± 0.02	1.70 ± 0.09
	17	1.93 ± 0.3	13.06 ± 1.6	800.68 ± 68.04	636.37 ± 60.14	2.74 ± 0.24	2.28 ± 0.15	0.34 ± 0.01	0.36 ± 0.01
	18	2.5 ± 0.25	24.76 ± 6.69	182.97 ± 5.25	350.57 ± 27.83	1.46 ± 0.09	2.30 ± 0.13	0.80 ± 0.05	0.66 ± 0.05
	19	3.11 ± 0.09	39.42 ± 0.84	92.58 ± 7.54	155.89 ± 13.61	1.68 ± 0.15	1.35 ± 0.06	1.82 ± 0.09	0.87 ± 0.07
14	20	4.29 ± 0.2	22.61 ± 1.95	83.40 ± 7.36	90.67 ± 7.32	1.30 ± 0.11	1.24 ± 0.11	1.55 ± 0.12	1.37 ± 0.13
	21	4.82 ± 0.14	19.58 ± 1.47	48.54 ± 2.98	42.40 ± 1.78	1.06 ± 0.09	1.01 ± 0.09	2.18 ± 0.11	2.38 ± 0.18
	22	5.29 ± 0.29	25.22 ± 1.82	119.49 ± 11.01	58.15 ± 4.22	0.98 ± 0.01	0.85 ± 0.05	0.82 ± 0.04	1.47 ± 0.12
	23	3.07 ± 0.05	14.45 ± 0.68	45.71 ± 3.46	38.96 ± 2.52	0.94 ± 0.06	0.86 ± 0.04	2.06 ± 0.02	2.26 ± 0.1
16	24	4.85 ± 0.28	41.5 ± 4.04	57.23 ± 4.17	46.60 ± 3.07	0.80 ± 0.03	0.81 ± 0.07	1.40 ± 0.08	1.73 ± 0.16
	25	4.21 ± 0.53	45.53 ± 4.06	35.34 ± 2.82	38.32 ± 2.05	0.75 ± 0.04	0.73 ± 0.03	2.13 ± 0.09	1.90 ± 0.13
	26	4.01 ± 0.69	31.78 ± 2.18	616.0 ± 35.1	534.87 ± 41.44	0.31 ± 0.01	0.26 ± 0.01	0.05 ± 0.01	0.05 ± 0.01
	27	2.91 ± 0.36	20.8 ± 1.4	92.80 ± 8.75	123.40 ± 10.06	1.35 ± 0.07	1.37 ± 0.11	1.46 ± 0.09	1.11 ± 0.1
Rat Aorta				91.13 ± 8.91	215.0 ± 18.9	5.45 ± 1.72	2.01 ± 0.17	5.98 ± 0.39	0.94 ± 0.08

## Data Availability

The data presented in this study are available within the article.
